# Restoration of Binocular Images Degraded by Optical Scattering through Estimation of Atmospheric Coefficients

**DOI:** 10.3390/s23218918

**Published:** 2023-11-02

**Authors:** Victor H. Diaz-Ramirez, Rigoberto Juarez-Salazar, Martin Gonzalez-Ruiz, Vincent Ademola Adeyemi

**Affiliations:** 1 Instituto Politécnico Nacional—CITEDI, Ave. Instituto Politécnico Nacional 1310, Tijuana 22310, Mexico; mruiz@citedi.mx (M.G.-R.); vademola@citedi.mx (V.A.A.); 2 CONAHCYT, Instituto Politécnico Nacional—CITEDI, Ave. Instituto Politécnico Nacional 1310, Tijuana 22310, Mexico; rjuarez@citedi.mx

**Keywords:** image dehazing, binocular vision, parameter estimation, optical scattering, image restoration

## Abstract

A binocular vision-based approach for the restoration of images captured in a scattering medium is presented. The scene depth is computed by triangulation using stereo matching. Next, the atmospheric parameters of the medium are determined with an introduced estimator based on the Monte Carlo method. Finally, image restoration is performed using an atmospheric optics model. The proposed approach effectively suppresses optical scattering effects without introducing noticeable artifacts in processed images. The accuracy of the proposed approach in the estimation of atmospheric parameters and image restoration is evaluated using synthetic hazy images constructed from a well-known database. The practical viability of our approach is also confirmed through a real experiment for depth estimation, atmospheric parameter estimation, and image restoration in a scattering medium. The results highlight the applicability of our approach in computer vision applications in challenging atmospheric conditions.

## 1. Introduction

Digital image processing allows the extraction of useful information from the real world by processing captured images of an observed scene [1]. In practice, image capturing can be affected by multiple perturbations, including additive noise, blurring, nonuniform illumination, and the effects of bad weather, among others [2]. In these conditions, the reliability of information extraction by image processing can be compromised [3].

Image restoration in the presence of optical scattering induced by haze is crucial for many real-world computer vision applications, such as autonomous driving [4], surveillance [5], and remote sensing [6], where accurate visual data are essential for decision-making and analysis. The development of effective techniques to mitigate the impact of scattering effects is critical for increasing the reliability of computer vision systems in real-world scenarios affected by adverse atmospheric conditions [7]. These techniques can have a direct impact on saving lives and increasing safety. In a scattering medium, image capturing is carried out in the presence of particles suspended in the medium, which produce a twofold undesired effect [8]. First, light from scene objects attenuates as the particle density in the medium and the distance of the scene points to the observer increase. Second, attenuated light is replaced by scattered light due to the interaction between the particles and the airlight. The problem of image restoration in these conditions is still open as it requires estimating several unknown components of the image formation process from one or more captured images. Furthermore, inherent space-variant scattering degradation makes conventional image restoration methods ineffective [9].

Currently, several approaches aim to improve the visibility of images degraded by optical scattering [10]. Table 1 presents widely investigated approaches for image restoration in these conditions. One approach utilizes different sensors to characterize the scene and scattering degradation [11,12,13,14]. This approach aims to simplify the image restoration problem. However, the time required to characterize the scattering degradation can be long because it is usually needed to wait for the atmospheric conditions to change. Another well-known approach to mitigate optical scattering effects consists of processing a single scene image [15,16,17,18,19]. This approach estimates the airlight and medium transmission function from a single hazy image. Next, a restored image is obtained using an atmospheric optics restoration model. This approach is very suitable for real-time applications. However, the image restoration problem becomes ill-posed due to the need to estimate several unknown image components from a single captured image. Consequently, the use of this approach often produces restored images with overprocessing effects and artificial artifacts that distort the original appearance and colors of the scene [7,15].

Because optical scattering degradation is space-variant, several stereo-vision-based methods have been proposed for image dehazing. One main advantage of these methods is their suitability for distinguishing between nearby slightly degraded scene objects and faraway highly degraded objects, enabling a reduction in overprocessing effects commonly produced by single-image dehazing methods. In this scenario, solving the image dehazing problem requires estimating the atmospheric parameters of the medium and scene depth involved in the image formation process. The successful single-image dehazing approach is unable to correctly estimate the unknown image formation components, providing only a partial solution for visibility improvement. Moreover, several stereo vision-based image dehazing methods rely on estimating the medium’s transmission function rather than the scene depth using complex machine learning models that require intensive training. The performance of these methods depends on the availability of a substantial dataset of hazy training images. Additionally, these methods are unsuitable for computer vision tasks involving metric distance calculations and three-dimensional reconstruction.

This work proposes a stereo vision approach for the accurate restoration of images degraded by optical scattering. This approach is based on estimating the scene depth and atmospheric parameters of the medium from a pair of binocular images degraded by optical scattering. The scene depth is obtained by triangulation using a disparity map computed through stereo matching [28]. Next, the atmospheric parameters of the medium are determined using an introduced robust estimator based on the Monte Carlo method. Finally, image restoration is performed using the estimated depth and atmospheric parameters in an optics-based restoration model. The proposed approach allows estimating the unknown components of an image-formation model based on atmospheric optics for scattering media. As a result, our approach accurately restores hazy images without intensive offline training. It is also well-suited for computer vision tasks involving metric distance computation and three-dimensional reconstruction.

This paper is organized as follows. Section 2 briefly describes different successful stereo-vision-based methods for image dehazing. Section 3 presents the proposed method for the accurate restoration of images degraded by optical scattering using binocular vision. The theoretical principles for estimating the scene’s depth in a scattering medium using binocular vision are presented. Additionally, we explain the proposed method for the estimation of the atmospheric parameters, namely, airlight and attenuation coefficients. Section 4 presents performance evaluation results obtained with the proposed method for restoring images degraded by optical scattering using test images from a well-known stereo image dataset. These results are also compared and discussed with those of two similar existing methods based on stereo vision. Moreover, the practical viability of the proposed approach is validated in a real laboratory experiment involving scene depth estimation, atmospheric parameter estimation, and image restoration in a scattering medium. Finally, Section 5 presents the conclusions of this research.

## 2. Related Works

In this section, we provide a brief overview of successful existing methods that utilize stereo vision for image dehazing. Murez et al. [22] proposed a photometric stereo-vision method for three-dimensional object reconstruction in scattering media. This approach models the scattered light as an unscattered point light source affected by a blurring degradation. A drawback of this method is that it is unsuitable for dynamic scene applications. Li et al. [23] proposed an iterative algorithm that performs both scene depth estimation and image dehazing using stereo vision. This method estimates the atmospheric parameters of the scene using a two-step procedure. First, the airlight coefficient is determined from the intensity distribution of the captured images. Then, the attenuation coefficient is estimated statistically. Fujimura et al. [24] proposed a deep-learning-based image dehazing cost volume method for multi-view stereo in scattering media. This method simultaneously estimates the scene depth, airlight, and attenuation coefficients from a set of captured stereo images. However, this method requires an intensive offline training process, and its performance depends upon the availability of a vast dataset of training images. Furthermore, this method requires a considerable number of images of the scene captured from different perspectives, which limits its applicability in dynamic scenarios. Recently, existing stereo-vision-based methods for image dehazing rely on estimating the medium transmission function rather than the scene depth through machine learning [25,26]. This approach is preferred over scene depth-based methods due to the compact dynamic range of the transmission function and the possibility of reducing the number of image components to be estimated, as both the depth and attenuation coefficient are considered in the transmission [29].

## 3. Image Restoration in a Scattering Medium Using Binocular Vision

**Figure 1:**
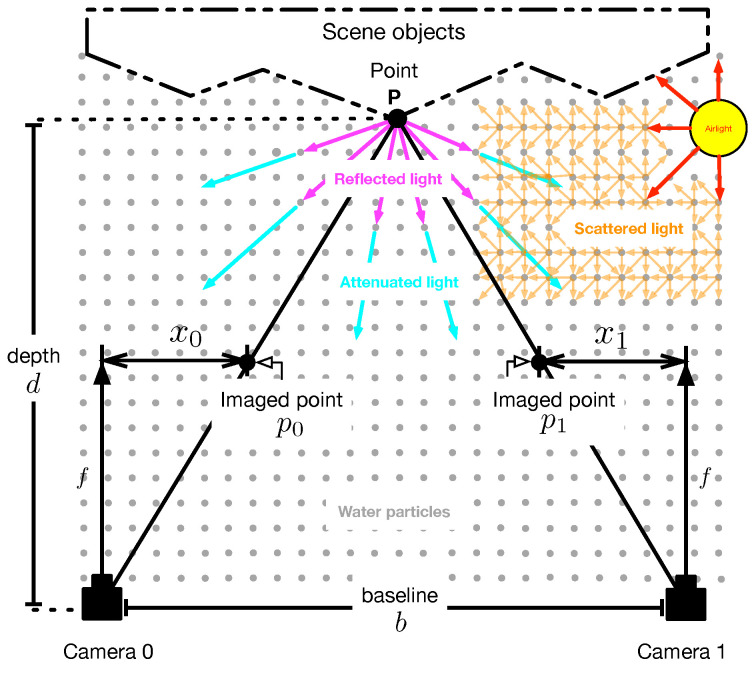
Geometry of a stereo vision system in a scattering medium. Reflected light (purple arrows) by scene objects is attenuated by scattering particles. The attenuated light (cyan arrows) is replaced by scattered light (orange arrows) due to airlight (red arrows).

Consider the binocular camera array that captures a pair of images of a scene in a homogeneous scattering medium, as shown in Figure 1. The scattering medium contains a density of suspended particles that attenuate the light reflected by objects in the scene, as the distance from the observer increases. In addition, the particles scatter the light from natural illumination (known as airlight), leading to a loss of visibility in the captured scene images. In this scenario, the *i*-th captured image of the scene can be given by [30]

(1) $$f_{i}\left(x,y\right)=s_{i}\left(x,y\right)\exp\left(-\beta d_{i}\left(x,y\right)\right)+A\left[1-\exp\left(-\beta d_{i}\left(x,y\right)\right)\right],$$

where $s_{i}\left(x,y\right)$ is the undegraded image captured by the left (i=0) or right (i=1) camera, $d_{i}\left(x,y\right)$ is the depth distribution of $s_{i}\left(x,y\right)$, β is an attenuation coefficient specifying the particle density, and *A* is an airlight coefficient [31]. From Equation (1), the image restoration can be performed as

(2) $$s_{i}\left(x,y\right)=f_{i}\left(x,y\right)\exp\left(\hat{\beta }{\hat{d}}_{i}\left(x,y\right)\right)+\hat{A}\left[1-\exp\left(\hat{\beta }{\hat{d}}_{i}\left(x,y\right)\right)\right],$$

where $\hat{\beta }$, $\hat{A}$, and ${\hat{d}}_{i}\left(x,y\right)$ are estimates of the unknown components of the image formation model given in Equation (1). In general terms, the estimation of these unknown components is hard because only one image equation per camera is available. This work presents the development of a robust and accurate method for estimating these unknown components from binocular images captured in a scattering medium for image restoration using Equation (2).

**Figure 2:**
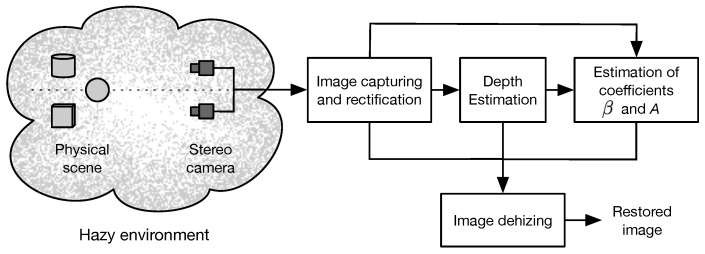
Block diagram of the proposed method for restoration of images degraded by optical scattering using binocular vision.

A suggested procedure for the restoration of images captured in a scattering medium is depicted in Figure 2. Initially, a pair of images of the scene is captured with a binocular camera array. Next, the captured images are rectified to meet the horizontal epipolar geometry [32]. The resultant rectified images are preprocessed to improve their visibility by applying a locally-adaptive contrast enhancement method [33]. Afterwards, the improved images are processed by stereo-matching to obtain estimates of the depth functions $d_{i}\left(x,y\right)$ of the scene by triangulation using the computed disparity map $\theta_{i}\left(x,y\right)$ [28]. Next, the hazy images $f_{i}\left(x,y\right)$, estimated depth $d_{i}\left(x,y\right)$, and disparity map $\theta_{i}\left(x,y\right)$ are used to estimate the atmospheric parameters β and *A* in a proposed algorithm based on the Monte Carlo method [34]. Finally, haze-free images of the scene are obtained using the restoration model given in Equation (2). In the next subsection, we explain in detail the proposed method for accurate estimation of the required components for the restoration model given in Equation (2).

### 3.1. Depth Estimation in a Scattering Medium

Let $P={\left[X,Y,Z\right]}^{T}$ be a point of a scene under the influence of a scattering medium that is imaged by a binocular camera array, as depicted in Figure 1. Let $p_{0}={\left[x_{0},y_{0}\right]}^{T}$ and $p_{1}={\left[x_{1},y_{1}\right]}^{T}$ be the pixel point of P in the image plane of the left and right camera, respectively, given as [32]

(3) $$\lambda_{i}H\left[p_{i}\right]=K_{i}L_{i}H\left[P\right],\;i=0,1,$$

where $\lambda_{i}$ are arbitrary scalar values, $K_{i}$ and $L_{i}$ are the intrinsic and extrinsic camera parameters, respectively, and for any vector x,

$$H_{w}\left[x\right]=\left[\begin{array}{c} x\\ w \end{array}\right]$$

is the homogeneous coordinate operator with base *w* [35]. For simplicity, we employ the pinhole camera model with intrinsic parameter matrix given as [35]

(4) $$K_{i}=\left[\begin{array}{ccc} k_{11} & k_{12} & k_{13}\\ 0 & k_{22} & k_{23}\\ 0 & 0 & 1 \end{array}\right]=\left[\begin{array}{ccc} f_{i}/\sigma_{x_{i}} & \zeta_{i} & \tau_{x_{i}}\\ 0 & f_{i}/\sigma_{y_{i}} & \tau_{y_{i}}\\ 0 & 0 & 1 \end{array}\right],$$

where $f_{i}$ is the camera’s lens focal length, $\zeta_{i}$ is the skewness, $\sigma_{x_{i}}\times \sigma_{y_{i}}$ specify the pixel size, and $(\tau_{x_{i}},$$\tau_{y_{i}})$ is the principal point, respectively, of the *i*-th camera. Without loss of generality, we consider that the world coordinate frame coincides with the local frame of the left camera (i=0). Therefore, the extrinsic parameters of the left and right cameras are

(5) $$\begin{array}{cc} L_{0} & =\left[\begin{array}{cc} I_{3}, & 0_{3} \end{array}\right], \end{array}$$

(6) $$\begin{array}{cc} L_{1} & =\left[\begin{array}{cc} R^{T}, & -R^{T}t \end{array}\right], \end{array}$$

where $I_{3}$ is the 3×3 identity matrix, $0_{3}$ is the 3×1 zero vector, R and t are a rotation matrix and a translation vector, respectively, which define the pose of the right camera with respect to the left camera. It is worth noting that rotation matrices will be handled using the Rodrigues formula

(7) $$R=I_{3}+\left(\sin\gamma_{1}\right)u^{\times }+\left(1-\cos\gamma_{1}\right)u^{\times }u^{\times },$$

where $\gamma_{1}$ is the rotation axis, u is a unit vector defining the rotation axis as

(8) $$u=\left[\begin{array}{c} u_{1}\\ u_{2}\\ u_{3} \end{array}\right]=\left[\begin{array}{c} \sin\gamma_{2}\cos\gamma_{3}\\ \sin\gamma_{2}\sin\gamma_{3}\\ \cos\gamma_{2} \end{array}\right],$$

where $\gamma_{2}$ and $\gamma_{3}$ are the polar and azimuth angles of vector u, and the superscript ${\left[\cdot \right]}^{\times }$ denotes the cross product operator as

(9) $$u^{\times }=\left[\begin{array}{ccc} 0 & -u_{3} & u_{2}\\ u_{3} & 0 & -u_{1}\\ -u_{2} & u_{1} & 0 \end{array}\right].$$

Note that the angles $\gamma_{1}$, $\gamma_{2}$, and $\gamma_{3}$ are sufficient to describe a rotation matrix using the Rodrigues formula given by Equation (7).

Now, let $\theta (p_{0})$ be the horizontal disparity value of the pixel point $p_{1}$ with respect to $p_{0}$. The point $p_{1}$ can be specified in terms of $p_{0}$ and $\theta (p_{0})$ as

(10) $$p_{1}=p_{0}+H_{0}\left[\theta \left(p_{0}\right)\right].$$

The spatial coordinates of the observed point P can be retrieved from Equations (3) and (10) by triangulation, solving the matrix equation

(11) $$\left[\begin{array}{ccc} K_{0}L_{0} & H_{1}\left[p_{0}\right] & 0_{3}\\ K_{1}L_{1} & 0_{3} & H_{1}\left[p_{0}+H_{0}\left[\theta \left(p_{0}\right)\right]\right] \end{array}\right]\left[\begin{array}{c} H_{1}\left[P\right]\\ \lambda_{0}\\ \lambda_{1} \end{array}\right]=0_{6},$$

where the singular value decomposition method can be used to efficiently compute the unknown vector ${\left[H_{1}{\left[P\right]}^{T},\lambda_{0},\lambda_{1}\right]}^{T}$ [36]. Finally, the required spatial coordinates are obtained as ${\left[X,Y,Z\right]}^{T}=H_{1}^{-1}\left[P\right]$.

#### Important Remarks

- The solution of Equation (11) requires prior calibration of the binocular system to determine the intrinsic and extrinsic camera parameters.
- The rotation matrix R and translation vector t in Equation (6) can be extracted from the fundamental matrix estimated during the image rectification process [32]. After the rectification, the extrinsic parameters of the right camera can be considered as
  (12) $$L_{1}=\left[I_{3}^{T},-t\right],\;\mathrm{with}\;t={\left[B,0,0\right]}^{T},$$
  where *B* is the stereo baseline.
- Although the scattering medium and scene depth are independent, the disparity estimation can be affected by the visibility reduction caused by the scattering degradation. To overcome this issue, we apply a locally adaptive contrast enhancement method to the captured hazy images [33].

### 3.2. Estimation of Atmospheric Parameters β and A

For simplicity, consider that $f_{i}$, $s_{i}$, and $d_{i}$ are column vectors containing the $N_{d}$ total pixel points of $f_{i}\left(x,y\right)$, $s_{i}\left(x,y\right)$, and $d_{i}\left(x,y\right)$, respectively, placed in a lexicographic order. Thus, considering the unknowns *A* and β as parameters, the *j*-th pixel of the *i*-th undegraded image can be expressed from Equation (2) as

(13) $$s_{i}\left[j;\beta ,A\right]=f_{i}\left[j\right]\exp\left(\beta d_{i}\left[j\right]\right)+A\left[1-\exp\left(\beta d_{i}\left[j\right]\right)\right].$$

Notice that by applying conventional algebraic manipulations, Equation (13) can be rewritten as

(14) $$\log\left(A-s_{i}\left[j;\beta \right]\right)=\beta d_{i}\left[j\right]+\log\left(A-f_{i}\left[j\right]\right).$$

Furthermore, assuming that the stereo images $s_{i}\left(x,y\right)$ are rectified and the scene depth $d_{i}\left(x,y\right)$ has been estimated as described in Section 3.1, the following assumptions on Equations (13) and (14) are valid:

1. $s_{0}\left[j;A\right]\approx s_{1}\left[j-\theta_{i}\left[j\right];A\right]$, where $\theta_{i}\left[j\right]$ is the *j*-th disparity value of the *i*-th image.
2. $\log\left(A-s_{0}\left[j;\beta \right]\right)\approx \log\left(A-s_{1}\left[j-\theta_{1}\left[j\right];\beta \right]\right)$.

To estimate β, let *b* be a random variable defined within the range $r_{\beta }=\left[\beta_{max}-\beta_{min}\right]$ of feasible values of the coefficient β. Thus, from Equation (13) and assumption 2, the coefficient β can be estimated by minimizing the following error function:

(15) $$\begin{array}{cc} \varepsilon^{2}\left(b\right) & ={\left[\log\left(A-s_{0}\left[j;b\right]\right)-\log\left(A-s_{1}\left[j-\theta_{i}\left[j\right];b\right]\right)\right]}^{2},\\ \; & ={\left[b\left(d_{0}\left[j\right]-d_{1}\left[j-\theta_{1}\left[j\right]\right]\right)+\log\left(A-f_{0}\left[j\right]\right)-\log\left(A-f_{1}\left[j-\theta_{1}\left[j\right]\right]\right)\right]}^{2}. \end{array}$$

It can be shown, that the random variable $b^{†}$ that minimizes Equation (15) can be obtained by solving $\frac{\partial }{\partial b}\left\{\varepsilon^{2}\left(b\right)\right\}=0$ as

(16) $$b^{†}=\frac{1}{\left(d_{1}\left[j-\theta_{1}\left[j\right]\right]-d_{0}\left[j\right]\right)}\log\left[\frac{A-f_{0}\left[j\right]}{A-f_{1}\left[j-\theta_{1}\left[j\right]\right]}\right];\;\left(d_{1}\left[j-\theta_{1}\left[j\right]\right]-d_{0}\left[j\right]\right)>0.$$

A reliable estimate of the unknown coefficient β can be obtained by computing the expected value of Equation (16) using the robust estimator for location given by [37]

(17) $$\hat{\beta }=E\left\{b^{†}\left[j\right]:b^{†}\left[j\right]>a\frac{b^{†}\left[j\right]-\mathrm{median}\left\{b^{†}\right\}}{\mathrm{MAD}\{b^{†}\}}\right\},$$

where

(18) $$\mathrm{MAD}\left\{b^{†}\right\}=1.4826\;\mathrm{median}\left\{b^{†}-\mathrm{median}\;\left\{b^{†}\right\}\right\},$$

is the median of absolute deviations from the median, and *a* is an outlier tolerance coefficient usually set to a=1.5.

Now, to estimate *A*, let α be a random variable within the range of feasible values of *A* defined as $r_{A}=\left[A_{max},A_{min}\right]$. By taking into account Equation (13) and assumption 1, the coefficient *A* can be estimated by minimizing the error function

(19) $$\varepsilon^{2}\left(\alpha \right)={\left(s_{0}\left[j;\alpha \right]-s_{1}\left[j-\theta \left[j\right];\alpha \right]\right)}^{2}.$$

The random variable $\alpha^{†}$, which minimizes Equation (19), can be obtained by solving $\frac{\partial }{\partial \alpha }\left\{\varepsilon^{2}\left(\alpha \right)\right\}=0$, as

(20) $$\alpha^{†}=\frac{f_{1}\left[j-\theta_{1}\left[j\right]\right]\exp\left(\beta d_{1}\left[j-\theta_{1}\left[j\right]\right]\right)-f_{0}\left[j\right]\exp\left(\beta d_{0}\left[j\right]\right)}{\exp\left(\beta d_{1}\left[j-\theta_{1}\left[j\right]\right]\right)-\exp\left(\beta d_{0}\left[j\right]\right)}.$$

An estimate of the airlight coefficient *A* can also be obtained by employing the robust location estimator given in Equations (17) and (18).

It is worth mentioning that in order to compute reliable estimates of β and *A*, it is required to solve the nonlinear system composed of Equations (16) and (20). There are different numerical approaches to solve this kind of system [38]. In this work, we propose a simple two-step approach based on the Monte Carlo method, whose steps are detailed in Algorithm 1.
**Algorithm 1:** Proposed algorithm for robust estimation of the airlight *A* and the attenuation coefficients β.
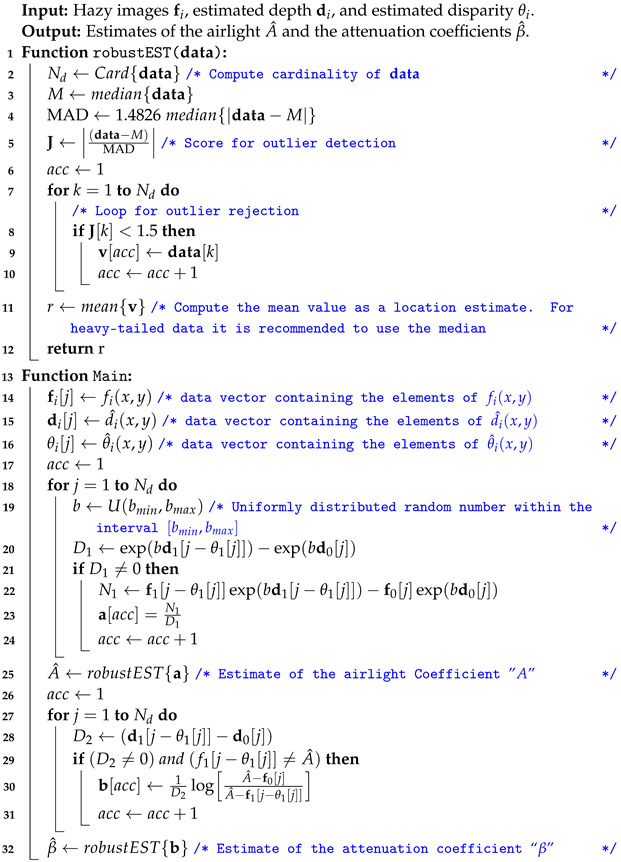


## 4. Results

In this section, the results obtained with the proposed approach for atmospheric parameter estimation and restoration of images degraded by optical scattering are presented and discussed. Initially, we briefly describe the dataset preparation for constructing a set of binocular images degraded by optical scattering. Afterwards, we present the performance evaluation results of the proposed method in estimating the atmospheric parameters β and *A*. For this, we analyze two cases: one assumes prior knowledge of the scene’s depth, while the other utilizes an estimate of the scene depth obtained from a disparity map computed by stereo matching from the input degraded images, as detailed in Section 3.1. Furthermore, the performance of the proposed method in the restoration of images degraded by optical scattering with different atmospheric parameter values is analyzed and discussed. Additionally, the performance of the proposed approach is compared with that of two existing stereo vision methods, namely, the method based on depth estimation proposed by Li et al. [23] and the method based on medium transmission estimation proposed by Ding et al. [25]. Finally, to validate the practical usefulness of the proposed approach, an experimental laboratory evaluation of scene depth estimation, atmospheric parameter estimation and image restoration of a scene is carried out in a real scattering medium.

### 4.1. Image Dataset Preparation

**Figure 3:**
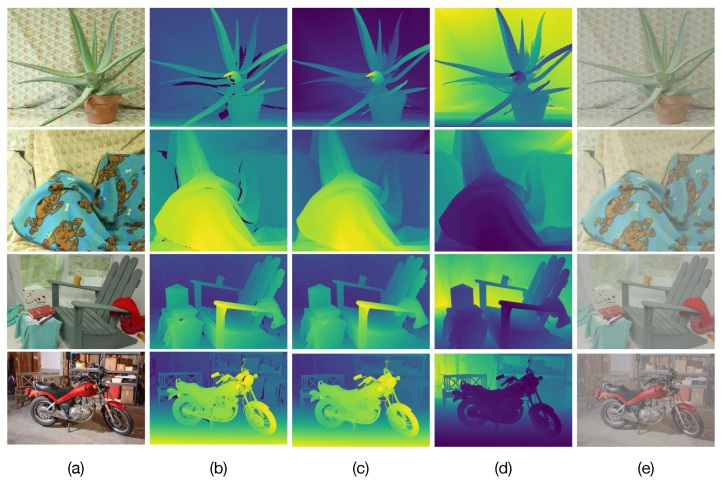
Examples of synthetic test images degraded by optical scattering. (**a**) Original images of the Middlebury stereo dataset. (**b**) Original ground-truth disparity map. (**c**) Refined ground-truth disparity map. (**d**) Computed scene depths. (**e**) Constructed test images degraded by optical scattering with A=187 and β=5.

We construct a test set of binocular images degraded by optical scattering using images of the well-known Middlebury stereo dataset [39,40,41]. This dataset contains several rectified stereo images and provides the corresponding ground-truth disparities. Figure 3a,b show examples of the dataset images and ground-truth disparities. Note that the disparity maps shown in Figure 3b contain dark color regions representing unknown disparities values.

The main challenge in the construction of synthetic images degraded by optical scattering lies in applying proper refinement techniques to remove these unknown values. This is because the unknown disparity values can lead to undesirable artifacts in the resulting synthetic images degraded by optical scattering and introduce errors in the estimation of atmospheric parameters as well as image restoration. To remove these unknown values, the ground-truth disparities are preprocessed with the hole-filling method presented in [28], obtaining refined disparities as shown in Figure 3c, where the unknown disparities are removed. Next, the undegraded binocular images of the dataset and their corresponding refined disparities are utilized to compute the scene depth by solving Equation (11) as detailed in Section 3.1, by considering the camera parameters specified by the Middlebury dataset. The computed depths for the images shown in Figure 3a are depicted in Figure 3d. Finally, test images degraded by optical scattering for prespecified values of the atmospheric parameters *A* and β are constructed using Equation (1) from the undegraded images and computed depth. Figure 3e presents examples of the constructed test images degraded by optical scattering from the undegraded images shown in Figure 3a.

### 4.2. Performance Evaluation in Estimation of the Atmospheric Parameters A and β

We evaluate the performance of Algorithm 1 in the estimation of the coefficients *A* and β from input binocular images degraded by optical scattering. First, we evaluate the performance of Algorithm 1 by assuming that the disparity map of each input binocular images is known. This test aims to quantify the performance of Algorithm 1 when the assumptions given in Section 3.2 are fully met. We also evaluate the performance of Algorithm 1 when the disparities and scene depth of each input image are estimated as described in Section 3.1. The disparities are computed using the stereo-matching method based on morphological correlation presented in Ref. [28]. Additionally, the performance of the existing stereo-vision methods proposed by Li et al. [23] and Ding et al. [25] are evaluated. For the method proposed by Li et al. [23], we consider the estimated disparities and scene depth utilized to evaluate the proposed method. For the evaluation of the method by Ding et al. [25], the value of β is obtained from the logarithm of the estimated transmission considering the ground-truth depth.

Twenty different binocular image pairs of the Middlebury dataset [39,40,41] are considered. For each image pair, we construct twenty-five image pairs degraded by optical scattering varying the atmospheric parameters within the range $r_{A}=\left[210,150\right]$ for *A* and $r_{\beta }=\left[5,1\right]$ for β. For each degraded image pair, the atmospheric parameters β and *A* are estimated with the two variants of Algorithm 1 and the existing stereo vision methods proposed by Li et al. [23] and Ding et al. [25]. The performance of parameter estimation is measured in terms of the mean-absolute-error (MAE) given as

(21) $$MAE=\frac{1}{N_{T}}\sum_{j=1}^{N_{T}}\left|v_{j}-{\hat{v}}_{j}\right|,$$

where $v_{j}$ is the real value, ${\hat{v}}_{j}$ is the estimated value, and $N_{T}=500$ is the total number of trials. Additionally, we compute the percentage of estimation accuracy (%Acc) as

(22) $$\%Acc=100\left(1-MAE/r\right),$$

where *r* is the parameter range.

**Figure 4:**
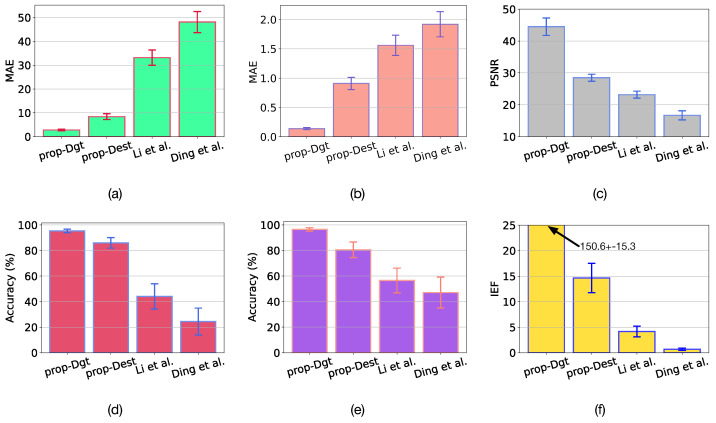
Results with 95% confidence of atmospheric parameter estimation and image restoration using the proposed method with ground-truth disparities (*prop-Dgt*), the proposed method with estimated disparities (*prop-Dest*), the method by Li et al. [23], and the method by Ding et al. [25]. (**a**) MAE in estimation of *A*. (**b**) MAE in estimation of β. (**c**) PSNR of image restoration. (**d**) %Acc in estimation of *A*. (**e**) %Acc in estimation of β. (**f**) IEF of image restoration.

The results of metrics MAE and %Acc with 95% confidence are presented in Figure 4a,b,d,e. The proposed algorithm yields very low MAE values in estimating both β and *A* coefficients when ground-truth disparities are utilized. This version of the proposed algorithm is referred to as *prop-Dgt*. It is worth mentioning that the estimation accuracy of the *prop-Dgt* is 95.31% for *A* and 96.5% for β. On the other hand, when the disparities are estimated by stereo-matching, the proposed algorithm obtains an estimation accuracy of 86% for *A* and 80.25% for β. This version of the proposed algorithm is referred to as *prop-Dest*. The accuracy reduction in *prop-Dest* is due to errors in disparity estimation. However, the incorporation of an advanced disparity refinement method [42] can help to increase the accuracy of the *prop-Dest* method. In contrast, the accuracy of parameter estimation obtained with the method proposed by Li et al. [23] was 44.68% for *A* and 54.5% for β, while the accuracy from the method by Ding et al. [25] was 24.4% for A and 47.08% for β. These results are considerably worse than those obtained with the proposed method.

### 4.3. Performance Evaluation in Restoration of Images Degraded by Optical Scattering

The estimated atmospheric parameters $\hat{\beta }$ and $\hat{A}$ using the proposed and existing methods for each pair of test images are used to perform image restoration with the help of Equation (2). The accuracy of image restoration is measured in terms of the peak signal-to-noise-ratio (PSNR) given as

(23) $$PSNR=20\log_{10}\left[\frac{MAX_{s}}{\sqrt{MSE}}\right],$$

where $MAX_{s}$ is the maximum intensity value of the reference image s(x,y), MSE is the mean-squared-error given as

(24) $$MSE=\frac{1}{N_{d}}\sum_{x}\sum_{y}{\left|s\left(x,y\right)-\hat{s}\left(x,y\right)\right|}^{2},$$

and $\hat{s}\left(x,y\right)$ is the restored image. Additionally, we compute the Image Enhancement Factor (IEF) given as

(25) $$IEF=\frac{\sum_{x}\sum_{y}{\left|f(x,y)-s(x,y)\right|}^{2}}{\sum_{x}\sum_{y}{\left|s\left(x,y\right)-\hat{s}\left(x,y\right)\right|}^{2}}.$$

**Figure 5:**
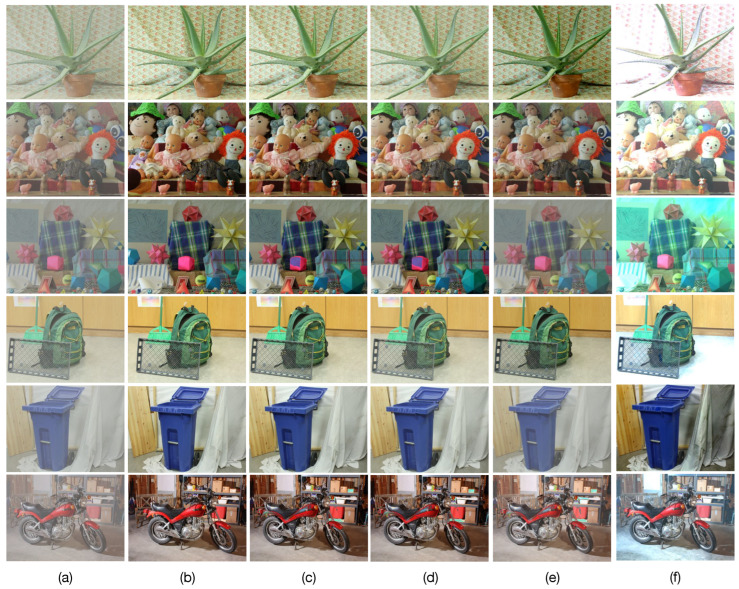
Image restoration results. (**a**) Input images degraded by optical scattering (A=190, β = 4.5). (**b**) Undegraded reference images. Restored images obtained by (**c**) the proposed method using ground-truth depth (*prop-Dgt*), (**d**) the proposed method using estimated depth (*prop-Dest*), (**e**) the method by Li et al. [23], and (**f**) the method by Ding et al. [25].

The obtained results are presented in the Figure 4c,f and Figure 5. In Figure 4c,f we can observe that when using the estimated atmospheric parameters with the *prop-Dgt* method, high PSNR and IEF values of 44.5±2.72 dB and 150.6±15.3, respectively, are obtained with 95% confidence. Figure 5c shows several restored images obtained with the *prop-Dgt* method. Note that these restored images closely match the reference undegraded images shown in Figure 5b. Now, note that the *prop-Dest* method produces a PSNR value of 28.48±1.06 dB and a IEF value of 14.65±5.4 with 95% confidence. Examples of the restored images obtained with the *prop-Dest* method are shown in Figure 5d. It can be seen that these restored images are very similar to the reference undegraded images shown in Figure 5b. In contrast, the stereo vision method proposed by Li et al. [23] yields a PSNR value of 23.1±1.16 dB and IEF value of 4.17±1.05, while the method proposed by Ding et al. [25] produces a PSNR value of 16.70±1.42 dB and IEF value of 0.6±0.23 with 95% confidence. The restored images obtained with the existing tested methods are shown in Figure 5e,f respectively. Note that some of these restored images contain very noticeable undesired effects. For instance, the Aloe image shown in Figure 5e contains overprocessing effects that distort the original colors of the undegraded image. Furthermore, in the Moebius and Recycle images, scattering effects still persist, reducing visibility in the restored image. These undesirable effects are caused by wrongly estimated atmospheric parameters by the Li et al. [23] method. Additionally, note that the method by Ding et al. [25] effectively removes the scattering degradation, but introduces undesirable over-processing effects, as shown in Figure 5f.

### 4.4. Performance Evaluation of the Proposed Method in a Real Scattering Medium

**Figure 6:**
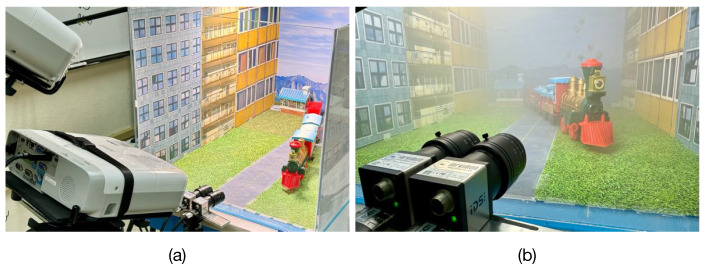
Constructed platform for experimental evaluation. (**a**) Setup for capturing the reference undegraded image of the scene and computation of the ground-truth depth using fringe projection profilometry. (**b**) Setup for image capturing of the scene in a real scattering medium.

The practical feasibility of the proposed method is validated in a real scattering medium. We constructed an experimental platform composed of an acrylic chamber with dimensions of 85×54×49 cm, containing a created scene and illuminated by an external light-emitting diode lamp, as depicted in Figure 6a. This platform permits capturing undegraded images of the scene, as shown in Figure 6a, and degraded scene images by introducing scattering particles into the chamber using a fog machine, as illustrated in Figure 6b. This experiment aims to evaluate the performance of the proposed image restoration method in real scattering conditions.

**Figure 7:**
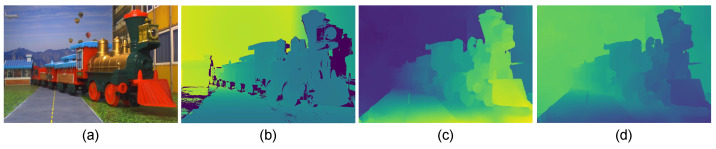
(**a**) Undegraded captured image of a real scene. (**b**) Ground-truth depth of the scene obtained by fringe projection profilometry. (**c**) Estimated disparity map of the scene in a scattering medium. (**d**) Depth of the scene computed by triangulation using the disparity map shown in (**c**).

First, undegraded images of the scene are captured using a binocular camera array when the chamber is free of scattering particles. The binocular camera array comprises two UI-3880CP-C-HQ R2, Imaging Development Systems, Obersulm, Germany, digital cameras with 3088×2076 pixels and 8 mm focal length imaging lens mounted on a horizontal fixture with a 36.42 mm baseline. The ground-truth depth of the scene is computed using three-dimensional spatial point computation by fringe projection profilometry [36]. The implemented fringe projection system consists of a binocular camera array and a pair of PowerLite W30, Epson, Suwa, Nagano, Japan, LCD projectors with a resolution of 1280×800 pixels, as depicted in Figure 6a. The intrinsic and extrinsic parameters of the cameras and projectors of the fringe projection system are determined using the calibration method proposed in Ref. [43]. The resultant estimated parameters are summarized in Table 2. Next, the captured images of the scene, denoted as ${\tilde{s}}_{0}\left(x,y\right)$ and ${\tilde{s}}_{1}\left(x,y\right)$, are rectified through the projective transformation method [32]. The rectified, undegraded image $s_{0}\left(x,y\right)$ and ground-truth depth $d_{0}\left(x,y\right)$ of the scene computed through fringe projection are presented in Figure 7a,b, respectively.

**Figure 8:**
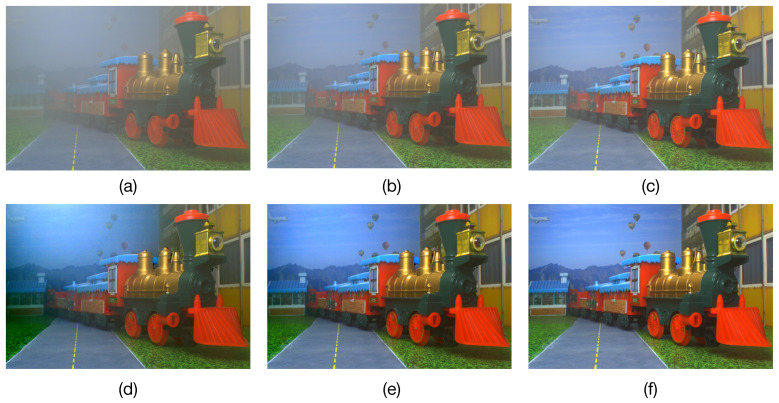
Captured images of the scene in the constructed platform in (**a**) severe scattering conditions, (**b**) moderate scattering conditions, and (**c**) mild scattering conditions. (**d**–**f**) Restored images corresponding to (**a**–**c**), respectively.

Afterwards, binocular images of the scene are captured with the used camera array by varying the concentration of scattering particles in the chamber. Figure 8a–c shows three captured images of the scene in severe, moderate, and mild scattering conditions, respectively. These captured images are assumed to be formed according to Equation (1). The proposed method depicted in Figure 2 is utilized for restoring the degraded images shown in Figure 8a–c. The degraded images are firstly preprocessed using the locally-adaptive filtering method suggested in Ref. [33]. Afterwards, the disparity map is computed utilizing the stereo-matching algorithm based on morphological correlation, as proposed in Ref. [28]. Finally, the disparity map is further refined using the algorithm suggested in Ref. [42]. The resultant disparity map of the scene computed under the influence of a scattering medium is shown in Figure 7c. Next, the scene’s depth is computed by solving Equation (11), considering the estimated disparity map and the camera parameters obtained by the calibration process given in Table 2. The computed depth of the scene is shown in Figure 7d. It can be observed that despite the presence of scattering particles in the medium, the estimated depth is closely approximated to the ground-truth depth shown in Figure 7b obtained by fringe projection profilometry.

Now, we employ the proposed Algorithm 1 to estimate the atmospheric parameters *A* and β for each degraded image shown in Figure 8a–c. The estimated atmospheric parameters for the three captured degraded images are presented in Table 3. It is worth noting that the estimated value of β increases with the concentration of scattering particles in the chamber while the estimated value of *A* decreases. The images of the scene captured in the presence of scattering particles depicted in Figure 8a–c are restored using the estimated atmospheric parameters and scene depth in the restoration model given in Equation (2). The resultant restored images are presented in Figure 8d–f. Note that all the restored images effectively suppress the effects of optical scattering and successfully restore the visibility of the scene without introducing noticeable artifacts or overprocessing effects. Furthermore, to assess the accuracy of the proposed method for image restoration in real optical scattering conditions, we calculate the PSNR and IEF values for each restored image shown in Figure 8d–f, considering the undegraded captured image shown in Figure 7a as the reference. The resultant PSNR and IEF values for each restored image are presented in the fourth and fifth column of Table 3. Note that the restored images produce PSNR values of 22.0 dB and 21.15 dB and IEF values of 1.42 and 1.21 for the mild and moderate scattering conditions, respectively. This result can also be confirmed by observing that the restored images shown in Figure 8e,f closely match the reference image shown in Figure 7a. Moreover, for the case of severe scattering degradation, the restored image yields a PSNR of 18.58 and IEF of 1.13 despite the fact that the light reflected by the farther objects in the scene is severely attenuated. These results confirm that the proposed method is highly effective in mitigating the effects of optical scattering and exhibits significant potential for computer vision applications including vehicle navigation and surveillance.

## 5. Conclusions

This research introduced a binocular vision-based method for restoring images captured in scattering media. This method performs scene depth estimation through stereo matching, estimation of atmospheric parameters, and image restoration based on atmospheric optics modeling. As a result, it effectively suppresses optical scattering effects in captured scene images without introducing noticeable artifacts in the restored images. The performance of the proposed approach was evaluated in terms of the accuracy of atmospheric parameter estimation and image restoration, using synthetic hazy images constructed from a well-known dataset. The proposed method outperformed two existing similar methods in all performed tests. To validate the practical viability of the proposed method, we performed a laboratory experiment comprising depth estimation, atmospheric parameter estimation, and image restoration, using binocular images captured in a real scattering medium. The results confirmed the effectiveness and robustness of the proposed method, highlighting its potential applicability for computer vision applications under challenging atmospheric conditions. A limitation of the proposed method is its exclusive design for homogeneous scattering media, potentially limiting its effectiveness in nonhomogeneous scattering conditions. Additionally, while the proposed method yields strong performance in scene depth estimation, errors in disparity estimation can affect the accuracy of atmospheric parameter estimation. These limitations can be addressed by considering an adaptive parameter estimation approach and advanced disparity refinement algorithms. For future work, we will explore the integration of machine learning techniques to adapt to different scattering conditions, implement the proposed approach in specialized hardware to enable massive parallelism, and assess its performance in real-world outdoor applications.

## Figures and Tables

**Table 1:** Summary of principal approaches for image restoration in scattering media.

Approach	Advantages	Disadvantages	References
Several sensors	- Accurate results. - Low overprocessing effects.	- Not feasible for dynamic scenes.	[11,12,13,14]
Single image	- Real-time operation. - Simple solution model.	- Introduction of noticeable artifacts. - Color distortion by overprocessing. - Poor performance due to unmet assumptions.	[15,16,17,18,20,21]
Stereo vision	- Accurate results. - Low overprocessing effects. - Feasible for 3D reconstruction.	- Operates with two or more images. - Susceptible to depth estimation errors. - Time-consuming.	[22,23,24,25,26,27]

**Table 2:** Estimated parameters from binocular and fringe projection system calibration.

Intrinsics:	$k_{11}$	$k_{12}$	$k_{13}$	$k_{22}$	$k_{23}$	
Camera-1	2.2279	−0.0163	0.0250	2.2231	−0.0191	
Camera-2	2.2493	−0.0135	0.0129	2.2479	0.0217	
Projector-1	2.5973	−0.0203	0.0250	2.5948	0.4871	
Projector-2	2.6106	−0.0200	0.0181	2.6071	0.4932	
**Extrinsics:**	$\gamma_{1}$	$\gamma_{2}$	$\gamma_{3}$	$t_{x}$	$t_{y}$	$t_{z}$
Camera-1	0	0	0	0	0	0
Camera-2	0.0203	1.0218	0.2878	36.4251	−0.6071	−0.4763
Projector-1	0.3635	1.5458	3.1202	−92.3790	−231.4493	−463.3557
Projector-2	0.2909	1.5772	2.2579	−238.3022	−31.7745	−315.9305

**Table 3:** Estimated atmospheric parameters and computed PSNR and IEF values in restoring the real images captured in a scattering medium shown in Figure 8a–c.

	Estimated Parameters			
Restored Scene	A	β	PSNR	IEF
mild scattering	170.4	3.41	22.0 dB	1.42
moderate scattering	161.3	5.37	21.15 dB	1.21
severe scattering	155.4	7.18	18.6 dB	1.13

## Data Availability

Publicly available datasets were analyzed in this study. These data can be found here: vision.middlebury.edu (accessed on 3 July 2023).

## Abbreviations

The following abbreviations are used in this manuscript:

*prop-Dgt*	Proposed algorithm Disparity ground-truth
*prop-Dest*	Proposed algorithm Disparity estimated
MAD	Median Absolute Deviation
MAE	Mean Absolute Error
%Acc	Percentage of estimation accuracy
PSNR	Peak Signal-to-noise-ratio
MSE	Mean Squared Error
IEF	Image Enhancement Factor
